# Developmental stages of *Sarcocystis* spp. in wild birds from Southeastern Brazil, with a review of Accipitriformes-associated species

**DOI:** 10.29374/2527-2179.bjvm006225

**Published:** 2026-02-18

**Authors:** Carlos Nei Ortúzar-Ferreira, Rodrigo Gredilha-Duarte, Gabriela de Carvalho Cid, Bruno Pereira Berto, Carlos Wilson Gomes Lopes

**Affiliations:** 1 Programa de Pós-Graduação em Biologia Animal, Instituto de Ciências Biológicas e da Saúde (ICBS), Universidade Federal Rural do Rio de Janeiro (UFRRJ). Seropédica, RJ, Brazil.; 2 Departamento de Saúde Coletiva, Universidade Federal do Estado do Rio de Janeiro. Rio de Janeiro, RJ, Brazil.; 3 Programa de Pós-Graduação em Medicina Veterinária, Instituto de Veterinária (IV), UFRRJ. Seropédica, RJ, Brazil.; 4 Laboratório de Biologia de Coccídios, Departamento de Biologia Animal, ICBS, UFRRJ. Seropédica, RJ, Brazil.; 5 Programa de Pós-Graduação em Ciências Veterinárias, Departamento de Parasitologia Animal, IV, UFRRJ. Seropédica, RJ, Brazil.

**Keywords:** coccidia, oocysts, bradyzoites, Accipitriformes, Passeriformes, coccídios, oocistos, bradizoítos, Accipitriformes, Passeriformes

## Abstract

The genus *Sarcocystis* comprises apicomplexan parasites associated with clinical manifestations, including reproductive and neurological disorders, in a wide range of domestic and wild animals. Infections by *Sarcocystis* spp. have been reported in birds on all continents except Antarctica. In this context, the present study reports two cases in which *Sarcocystis* spp. were identified at different developmental stages, endogenous and exogenous, in wild birds captured in Southeastern Brazil. Oocysts and sporocysts were detected in the feces of a roadside hawk *Rupornis magnirostris*, captured on Marambaia Island, Rio de Janeiro State, Brazil. In addition, bradyzoite cysts were observed in the muscle tissue of a rufous-bellied thrush *Turdus rufiventris*, captured in Itatiaia National Park. Accordingly, this study provides comments on the parasitic dynamics of *Sarcocystis* spp. in a wild environment and discusses the potential species involved in these host-parasite associations. For *Sarcocystis* sp. detected in the rufous-bellied thrush, based on evidence from previous studies, we strongly suggest that this species is *Sarcocystis falcatula*. In contrast, for *Sarcocystis* sp. detected from the roadside hawk, no specific species assignment can be made, as data on hawks acting as definitive hosts of *Sarcocystis* spp. in South America remain scarce. Although *S. falcatula* may involve Accipitriformes in its life cycle, members of this order are considered intermediate rather than definitive hosts for this species. Therefore, we present herein a survey of *Sarcocystis* spp. that involve Accipitriformes in their life cycles, including comparative data on sporocyst measurements for each species.

## Resumo

O gênero *Sarcocystis* compreende parasitos apicomplexos associados a manifestações clínicas, incluindo distúrbios reprodutivos e neurológicos, em uma ampla gama de animais domésticos e silvestres. Infecções por *Sarcocystis* spp. foram relatadas em aves em todos os continentes, com exceção da Antártica. Nesse contexto, o presente estudo relata dois casos nos quais *Sarcocystis* spp. foram identificados em diferentes estágios de desenvolvimento, endógeno e exógeno, em aves silvestres capturadas no Sudeste do Brasil. Oocistos e esporocistos foram detectados nas fezes de um gavião-carijó *Rupornis magnirostris*, capturado na Ilha da Marambaia, estado do Rio de Janeiro, Brasil. Além disso, cistos contendo bradizoítos foram observados em tecido muscular de um sabiá-laranjeira *Turdus rufiventris*, capturado no Parque Nacional do Itatiaia. Dessa forma, este estudo apresenta considerações sobre a dinâmica parasitária de *Sarcocystis* spp. em ambiente silvestre e discute as possíveis espécies envolvidas nessas associações hospedeiro-parasito. Para *Sarcocystis* sp. detectada no sabiá-laranjeira, com base em evidências de estudos prévios, sugerimos fortemente que essa espécie corresponda a *Sarcocystis falcatula*. Em contraste, para *Sarcocystis* sp. detectada no gavião-carijó, não é possível atribuir uma espécie específica, uma vez que os dados sobre gaviões atuando como hospedeiros definitivos de *Sarcocystis* spp. na América do Sul ainda são escassos. Embora *S. falcatula* possa envolver representantes da ordem Accipitriformes em seu ciclo de vida, esses atuam como hospedeiros intermediários, e não definitivos, para essa espécie. Portanto, apresentamos aqui um levantamento das espécies de *Sarcocystis* que envolvem Accipitriformes em seus ciclos de vida, incluindo dados comparativos das medidas dos esporocistos de cada espécie.

## Introduction

*Sarcocystis* comprises a diverse group of cyst-forming coccidian parasites (Sarcocystidae) characterized by a heteroxenous life cycle (Fayer, 1980; Gardiner et al., 1998; Atkinson et al., 2008). The genus currently includes approximately 220 described species, and in about 10% of these, birds of prey act as definitive hosts (Juozaitytė-Ngugu et al., 2025). However, Dubey et al. (2015) pointed out that the complete life cycle is known for only 26 species. Most species have been described solely from their intermediate hosts, and fewer than half have a known definitive host. Moreover, in comparison with other vertebrate groups, such as mammals, birds remain relatively underinvestigated as either intermediate or definitive hosts of *Sarcocystis* spp. (Prakas & Butkauskas, 2012; Llano et al. 2022).

Globally, numerous avian species serve as intermediate hosts, harboring characteristic intramuscular tissue cysts that must be ingested by definitive hosts to complete transmission (Box & Smith, 1982; Černá, 1984; Dubey et al., 2004; Kutkienė et al., 2012; Berto et al., 2014). Avian intermediate hosts become infected via the fecal-oral route through the ingestion of oocysts shed by definitive hosts into the environment (Fayer, 1980; Gardiner et al., 1998). Accordingly, species of this genus can be taxonomically identified based on the presence of septate polyzoic cysts in intermediate hosts and/or oocysts and sporocysts recovered from the feces of definitive hosts (Markus et al., 1974; Taylor et al., 2017).

In this context, the present study reports two cases in which *Sarcocystis* spp. were identified at different developmental stages, endogenous and exogenous, in wild birds captured in Southeastern Brazil. The first case concerns a definitive host, as oocysts and sporocysts of a *Sarcocystis* sp. were recovered from fecal samples of a roadside hawk *Rupornis magnirostris*. The second case corresponds to the identification of a *Sarcocystis* sp. in an intermediate host, since bradyzoite cysts were observed in the muscle tissue of a rufous-bellied thrush *Turdus rufiventris*.

Studies addressing sarcocystosis in Neotropical wild birds remain scarce. Therefore, this study provides comments on the parasitic dynamics of *Sarcocystis* spp. in a wild environment and discusses the potential species that may occur in these hosts.

## Material and methods

### Sampling area

**Figure 1:**
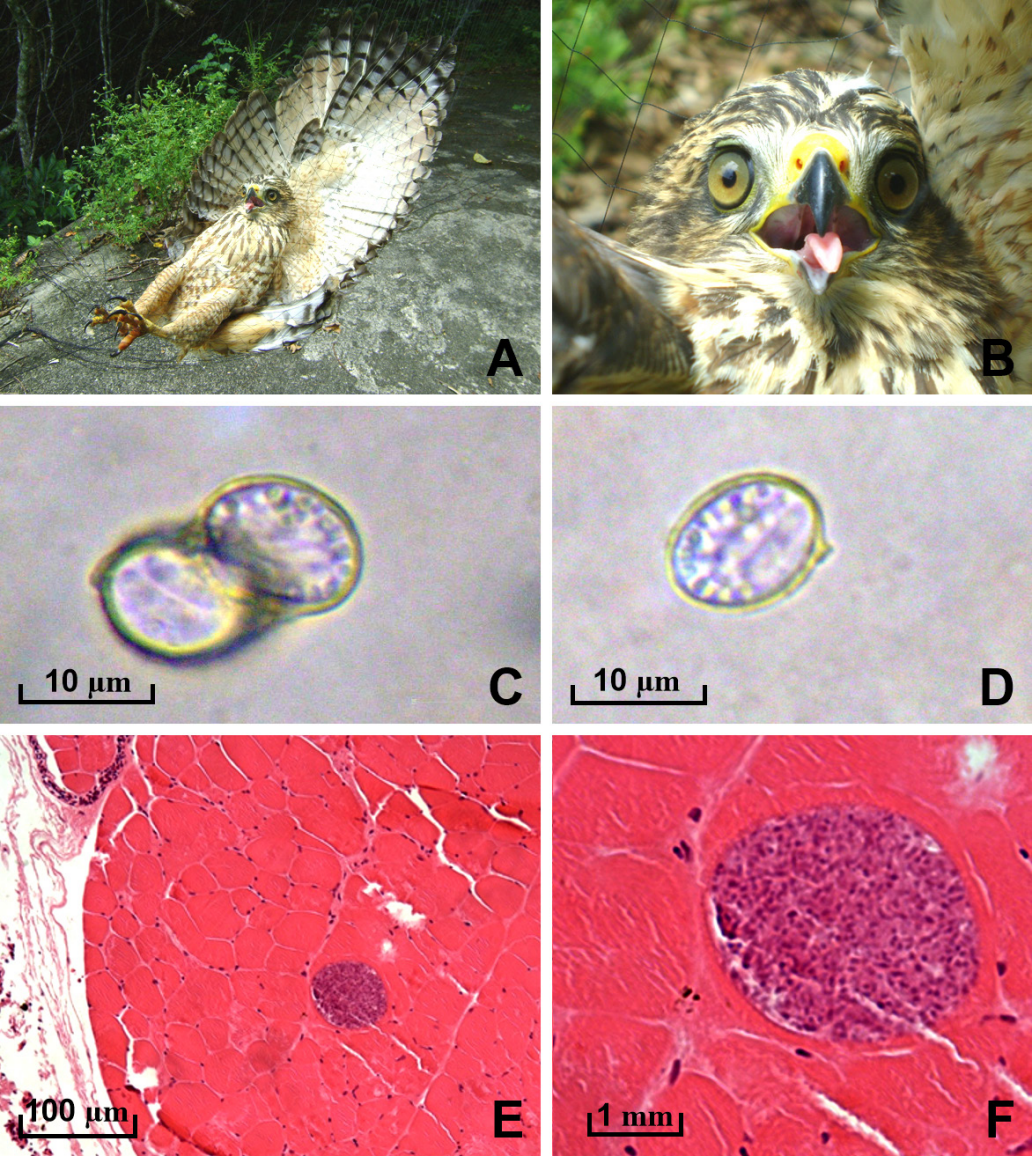
A roadside hawk *Rupornis magnirostris* captured in mist net in Marambaia Island, Southeastern Brazil (A and B); Photomicrographs of an oocyst (C) and a sporocyst (D) of *Sarcocystis* sp. recovered from the roadside hawk; photomicrographs of a histological section showing the presence of bradyzoite cysts of a *Sarcocystis* sp. in pectoral muscles of a rufous-bellied thrush *Turdus rufiventris* captured in the Itatiaia National Park, Southeastern Brazil (E and F).

These reports arose from incidental findings obtained during fieldwork conducted as part of independent research projects with different primary objectives. The first project aimed to survey the diversity of eimeriid coccidian parasites in wild birds on Marambaia Island, Southeastern Brazil (23°03′38.86″S, 43°58′47.56″W). During this survey, one roadside hawk *R. magnirostris*, and one white-tipped dove *Leptotila verreauxi*, were captured in September 2008 (Figure 1A, B). Notably, the hawk was captured after attempting to prey upon the dove, which had already been caught in a mist net. The second project focused on the molecular characterization and phylogenetic analysis of hemoparasites in wild birds from Itatiaia National Park, Southeastern Brazil (22°22′8.36″S, 44°44′39.27″W). Within this context, one rufous-bellied thrush *T. rufiventris*, was captured in September 2017.

### Capture method

Wild birds in both projects were captured using ornithological mist nets manufactured by Ecotone® (model 716/12; dimensions 12 × 2.5 m; mesh size 16 mm), which were deployed in the forest understory for a period of 10 hours beginning at dawn. The nets were mounted on aluminum poles up to 3 m in height and positioned 10–20 cm above ground level. Nets were inspected at 15-minute intervals to remove captured birds. Birds were identified to species level using standard field guides (Ridgely & Tudor, 2009; Sigrist, 2014). Photographs were taken to confirm identifications, and all captured individuals were subsequently measured and weighed.

### Ethics and permits

In the aforementioned projects, all captured birds were released after the collection of samples relevant to each study (fecal and blood samples). However, despite adherence to established capture protocols and animal welfare procedures, the rufous-bellied thrush died as a result of stress associated with capture and handling. Although undesirable, such events may occasionally occur, and there are reports of non-negligible mortality rates associated with bird handling, particularly due to capture myopathy (Ward et al., 2011). Following death, the bird was kept at approximately 4 °C and subsequently transported to the Veterinary Institute (Instituto de Veterinária – IV) of the Federal Rural University of Rio de Janeiro (Universidade Federal Rural do Rio de Janeiro – UFRRJ) for necropsy. All procedures were conducted under permits issued by the Chico Mendes Institute for Biodiversity Conservation (Instituto Chico Mendes de Conservação da Biodiversidade – ICMBio) through the Biodiversity Authorization and Information System (Sistema de Autorização e Informação em Biodiversidade – SISBIO), license no. 55195, and were approved by the Animal Ethics Committee (Comitê de Ética no Uso de Animais – CEUA) of the UFRRJ, under protocol no. IV-6121130617.

### Laboratory analyses

Fecal samples collected from the hawk were individually placed in centrifuge tubes containing 2.5% potassium dichromate solution (K_2_Cr_2_O_7_) and examined at the Laboratory of Biology of Coccidians (Laboratório de Biologia de Coccídios – LABICOC), UFRRJ. Samples were incubated at room temperature (25 °C) for 7 days to allow sporulation (Dolnik, 2006). Oocysts and sporocysts were isolated by flotation in saturated Sheather’s sugar solution (specific gravity: 1.20) and examined microscopically following the technique described by Duszynski and Wilber (1997). Morphological observations, photomicrography, and measurements were performed using an Olympus BX binocular microscope (Olympus Optical, Tokyo, Japan) equipped with a Eurekam 5.0 digital camera (BEL Photonics, Monza, Italy).

The deceased thrush was processed at the Histopathology Laboratory (Laboratório de Histopatologia) of the Department of Pathological Anatomy (Departamento de Anatomia Patológica), UFRRJ. Tissue fragments from the lungs, liver, brain, spleen, heart, and pectoral muscle were fixed in 10% buffered formalin, embedded in paraffin, sectioned at 5.0 μm, and stained with hematoxylin and eosin (H&E). Slides were examined under light microscopy, and morphometric measurements were obtained digitally using an Olympus DP73 camera.

## Results

Exogenous and endogenous stages of *Sarcocystis* spp. were observed in the hawk and the thrush, respectively. Coproparasitological examination using Sheather’s flotation method revealed the presence of oocysts and sporocysts in fecal samples collected from the hawk (Figure 1C, D). In the thrush, histopathological examination demonstrated septate cystic structures containing bradyzoites of *Sarcocystis* sp. within the pectoral muscle, randomly distributed among skeletal muscle fibers (Figure 1E, F).

Species-level identification was not possible based solely on morphological characteristics. Comprehensive elucidation of biological aspects, including the definitive and intermediate hosts involved in each case, is required and should be complemented by detailed morphometric and molecular analyses to enable reliable species identification.

## Discussion

The genus *Sarcocystis* comprises numerous species described across a wide range of animal taxa, including birds (Taylor et al., 2017; Votýpka et al., 2016). According to Odening (1998), 12 *Sarcocystis* spp. have birds as definitive hosts, whereas 20 *Sarcocystis* spp. have birds as intermediate hosts; among these, two species have been reported to use birds as both definitive and intermediate hosts. Kutkienė et al. (2012) documented nearly 30 *Sarcocystis* spp. infecting the muscle tissues of birds representing at least 13 avian orders. To date, one of the most comprehensive investigations focusing on birds from South America is that of Llano et al. (2022), in which the authors evaluated the skeletal striated pectoral muscle of 400 birds belonging to various orders in Brazil and detected *Sarcocystis* infection in 38 individuals using molecular methods.

Based on the predation behavior observed in the present study, specifically a roadside hawk preying upon a white-tipped dove that had been captured in a mist net, certain *Sarcocystis* spp. are particularly noteworthy because their life cycles involve Columbiformes as intermediate hosts and Accipitriformes as definitive hosts. *Sarcocystis calchasi* circulates between the Northern goshawk *Accipiter gentilis* as the definitive host and the domestic pigeon *Columba livia* as the intermediate host (Olias et al., 2010a). *Sarcocystis columbae* has the wood pigeon *Columba palumbus* as its intermediate host, although the definitive host remained unknown at the time of its description. Subsequently, the Eurasian sparrowhawk *Accipiter nisus* and *A. gentilis* were confirmed as definitive hosts for both species (Olias et al., 2011; Mayr et al., 2016; Prakas et al., 2020a).

With regard to the host genus *Turdus*, which tested positive for sarcocysts in the present study, a report from Lithuania led to the description of *Sarcocystis turdusi* (Kutkienė et al., 2012). Subsequent studies demonstrated that *Accipiter* spp. hawks act as definitive hosts for this species (Prakas et al., 2020a).

*Sarcocystis* infections are often asymptomatic; however, several studies have highlighted their occurrence in both free-ranging and captive birds as an important epidemiological indicator of environmental imbalance. These studies also emphasize the increased risks faced by species within the orders Psittaciformes, Columbiformes, and Passeriformes, which are particularly vulnerable to trafficking and extinction (Smith et al., 1990; Godoy et al., 2009). In this context, *S. falcatula*, *Sarcocystis lindsayi*, and *S. calchasi* deserve special attention. Infections caused by *S. falcatula* and *S. lindsayi* are associated with hyperacute clinical manifestations and severe pulmonary damage, whereas *S. calchasi* is known to cause meningoencephalitis (Dubey et al., 2015).

Furthermore, some *Sarcocystis* spp., such as *Sarcocystis neurona* and *S. falcatula*, exhibit multi-host transmission, infecting a broad range of intermediate and incidental hosts (Olias et al., 2010b). *Sarcocystis falcatula* is among the most prevalent species in the Americas, with intermediate hosts spanning several avian orders, including Accipitriformes, Columbiformes, and Passeriformes (Llano et al., 2022). Notably, *S. falcatula* can be regarded as a species complex, with two genetic lineages described in the Americas: one restricted to North America and another circulating in birds throughout the continent (Llano et al., 2022). In this context, Mayr et al. (2016) and Šukytė et al. (2023) highlighted the high diversity of *Sarcocystis* spp. infecting hawks and other predatory birds, suggesting the existence of additional, yet undescribed, species in South America.

In a Brazilian study, *S. falcatula* was identified in 14 bird species, including pigeons and hawks, revealing high genetic diversity of this parasite in South America. Other species, such as *Sarcocystis halieti* and *S. lindsayi*, were also detected in birds, with opossums of the genus *Didelphis* acting as definitive hosts (Llano et al., 2022; Dubey et al., 2001; Stabenow et al., 2008; Stabenow et al., 2012). Considering the findings of Llano et al. (2022), it is plausible that the species observed in *T. rufiventris* in the present study corresponds to *S. falcatula*, given that this species has been reported from several Passeriformes families, including Vireonidae, Thraupidae, and Icteridae. In contrast, the species detected in *R. magnirostris* remains uncertain, as definitive identification is precluded by the morphological nonspecificity of *Sarcocystis* oocysts. As emphasized by the aforementioned authors, relatively few studies have assessed the parasitic diversity of this genus in wild animals from South America.

It is important to emphasize that species-level identification of *Sarcocystis* spp. cannot be achieved solely on the basis of cyst or oocyst morphology. Oocysts are morphologically uniform and do not present taxonomic characters such as those used for eimeriid coccidia, and the ultrastructure of the cyst wall alone is insufficient to discriminate among species. For instance, *S. calchasi* and *S. columbae*, both of which parasitize pigeons, form very similar cysts; nevertheless, molecular analyses have demonstrated that they are distinct species (Olias et al., 2010a,b; Llano et al. 2022). Therefore, ultrastructural characterization using electron microscopy (Stabenow et al., 2012), together with life cycle studies and genetic analyses, is essential for the accurate identification or description of new *Sarcocystis* species. Unfortunately, these approaches were not applied in the present study, and thus the parasite could only be identified at the generic level.

Another genus of Sarcocystinae associated with birds of prey as definitive hosts is *Frenkelia* (Upton & McKown, 1992). This genus has been taxonomically distinguished from *Sarcocystis* by the presence of cysts restricted exclusively to the nervous tissue of rodents, which act as obligate intermediate hosts (Smith, 1981; Long, 1990; Upton & McKown, 1992; Baker, 2006). The morphology of the cysts has traditionally been used to differentiate between the two recognized species of *Frenkelia*: *Frenkelia microti*, which forms lobulated cysts, and *Frenkelia glareoli*, which forms rounded cysts (Modrý et al., 2004). A third species, *Frenkelia clethrionomyobuteonis*, was later described but subsequently synonymized with *F. glareoli* (Odening, 1998).

An understanding of parasitic dynamics and ecological interactions is essential for the identification of coccidia belonging to the family Sarcocystidae, as many species are morphologically indistinguishable. For example, Fayer (1981) noted that the oocysts of *Toxoplasma gondii* and *Hammondia hammondi* are remarkably similar; both species share rodents as intermediate hosts and cats as definitive hosts. However, *H. hammondi* is obligatorily heteroxenous, such that infection in mice occurs only via ingestion of oocysts (and not tachyzoites or bradyzoites), and infection in cats occurs only via ingestion of tissue cysts, in contrast to the ecological plasticity observed in the life cycle of *T. gondii* (Frenkel & Dubey, 2000). Moreover, *H. hammondi* has a more restricted host range, is less pathogenic, and forms cysts exclusively in skeletal muscle, whereas *T. gondii* forms cysts in a wide variety of cell types (Smith, 1981; Baker, 2006).

Accordingly, ecological information, particularly data on host associations and the parasitic stages involved in the life cycle, can be critical for resolving taxonomic uncertainties. Because *Frenkelia* has not been reported to use Passeriformes as intermediate hosts, and because the cyst observed in *T. rufiventris* was located in striated skeletal muscle, the available evidence strongly supports its identification as *Sarcocystis*. With regard to the findings in the hawk, the sporocysts observed could theoretically be attributed to *Frenkelia*, since *F. microti* predominantly uses hawks of the genera *Buteo* and *Rupornis* as definitive hosts (Upton & McKown, 1992). However, it should be noted that *Frenkelia* was proposed as a junior synonym of *Sarcocystis* by Mugridge et al. (1999) and Modrý et al. (2004), although this taxonomic interpretation has not been universally accepted. Nevertheless, in the present study, both findings, in the thrush and in the hawk, are treated as *Sarcocystis* spp., as phylogenetic analyses do not consistently support the separation of these genera. Moreover, as the life cycle of *Sarcocystis* has become better understood, it has been demonstrated that some species (e.g., *S. neurona*) are capable of forming sarcocysts in the brains of their hosts, further weakening the ecological and biological distinctions historically used to justify generic separation (Verma et al., 2017a).

The advent of molecular techniques has undoubtedly enabled more precise taxonomic differentiation among cryptic coccidian groups, and all of the genera discussed here can be distinguished using molecular tools. Nevertheless, it must be emphasized that the isolated use of molecular data can also be misleading. For instance, *T. gondii* exhibits up to 3% genotypic variation among lineages, with population genetic studies revealing complex geographic patterns. Strains from North America and Europe largely comprise three closely related clonal lineages, with a fourth variant more commonly detected in wild animals, whereas South American strains are more genetically diverse and appear largely isolated from those in the Northern Hemisphere (Su et al., 2012; Khan et al., 2014). Despite this diversity, *T. gondii* has not been subdivided into multiple species. Conversely, *Isospora* spp. have been separated based on differences as small as 0.6% for the 18S gene and 1.7% for the COI gene, even in the absence of clear morphological differentiation (Hafeez et al., 2014).

Another relevant consideration is that, in organism groups with limited genetic characterization (i.e., few sequenced loci), molecular identification is constrained because it relies heavily on comparisons with sequences already available in public databases. Most *Sarcocystis* species have at least one sequenced locus, usually the 18S rDNA, which is highly conserved and facilitates primer design for related taxa but is suboptimal for species delimitation. For *Sarcocystis* spp., the ITS1 region is considered more informative Llano et al. (2022). Historically, reliance on the 18S gene led to *S. neurona* and *S. falcatula* being considered synonymous, as no substantial differences were detected at this locus (Fenger et al., 1995; Dame et al., 1995; Dubey et al., 2015). As emphasized by Llano et al. (2022), 18S rDNA analysis alone is insufficient for reliable species discrimination within *Sarcocystis*. Therefore, the most robust approach involves concatenated analyses using multiple loci, preferably those with lower levels of conservation.

Finally, the present study provides a comparative morphometric table of the sporocysts observed herein alongside those of *Sarcocystis* species (and the synonymous *Frenkelia*) described to date in Accipitriformes (Table 1), with the aim of supporting future research on this group of parasites. Many *Sarcocystis* species have been described solely on the basis of cysts in intermediate hosts, leaving critical information about definitive hosts and sporocyst morphology unknown. It is also noteworthy that some species use birds of prey as intermediate rather than definitive hosts, a pattern that may initially seem counterintuitive given predator-prey relationships (Odening, 1998; Wünschmann et al., 2010; Llano et al., 2022). Nonetheless, this highlights the dynamic and often unexpected nature of trophic interactions in natural systems, and illustrates how parasites exploit diverse ecological opportunities to complete their life cycles.

**Table 1:** Comparative morphometrics of sporocysts of *Sarcocystis* spp. recorded from birds of the order Accipitriformes.

Coccidia	Intermediare host	Definitive host	Sporocysts			References
			Length (µm)	Width (µm)	L/W ratio	
*Sarcocystis jaypeedubeyi* (syn. *Frenkelia microti*, *Isospora buteonis*, *Sarcocystis buteonis*, *Toxoplasma microti, Toxoplasma glareolus*)	Rodents from the families Cricetidae, Muridae, Chinchillidae, Erethizontidae, Leporidae. [*Clethrionomys glareolus*, *Clethrionomys rufocanus bedfordiae*, *Cricetus cricetus*, *Lemmus lemmus*, *Mesocricetus auratus*, *Microtus agrestis*, *Microtus arvalis*, *Microtus modestus*, *Microtus ochrogaster*, *Ondatra zibethica*, *Apodemus agrarius*, *Apodemus flavicollis*, *Apodemus sylvaticus*, *Mastomys natalensis*, *Mus musculus*, *Rattus norvegicus*, *Chinchilla laniger*, *Erethizon dorsatum*, *Oryctolagus cuniculus* (some of them experimentally)].	*Buteo buteo*, *Buteo borealis*, *Buteo swainsoni*, *Buteo jamaicensis, Accipiter cooperi, Accipiter gentilis, Accipiter nisus, Asio flammeus*	11.7±14.6 (12.2 μm)	8.7±12.0 (9.9 μm)	not reported	Upton et al. (1990), Odening (1998); Mugridge et al. (1999), Fichet-Calvet et al. (2004), Modrý et al. (2004), Mayr et al. (2016), Verma et al. (2017a), Prakas et al. (2024c)
*Sarcocystis glareoli* (syn. *Frenkelia glareoli*, *Frenkelia clethrionomyobuteonis, Frenkelia buteonis, Toxoplasma glareoli*)	Rodents of Cricetidae Family [*Arvicola sapidus*, *Arvicola amphibius, Arvicola terrestris C. glareolus* (also experimentally), *C. rufocanus*, *Clethrionomys rutilus, A. sylvaticus, M. arvalis, M. agrestis*]	*B. buteo, Buteo lagopus* (experimentally), *Buteo lineatus, B. jamaicensis, Falco tinnunculus, A. gentilis, Gyps fulvus, A. nisus, Milvus milvus,*	11.3±13.8 (12.5 μm)	7.8±10.0 (8.8 μm)	not reported	Rommel et al. (1977), Upton et al. (1990), Odening (1998), Mugridge et al. (1999), Fichet-Calvet et al. (2004), Prakas et al. (2024b, c), Juozaitytė-Ngugu et al. (2025)
*Sarcocystis citellibuteonis*	*Citellus fulvus*	*B. buteo*	11,9±13,3 (12.6 μm)	9,8±10,5 (9.8 μm)	not reported	Pak et al. (1989a), Odening (1998)
*Sarcocystis jamaicensis*	Experimental intermediate host: IFN-c gene knockout mouse	*B. jamaicensis*	11.2±13.7 (12.5 μm)	8.8±10.9 (9.9 μm)	not reported	Verma et al. (2017)
*Sarcocystis strixi*	Experimental intermediate host: Interferon Gamma Gene Knockout Mice, *A. flavicollis*	*Strix varia, Bubo bubo, Milvus migrans, M. milvus, B. buteo, F. tinnunculus, Falco naumanni, G. fulvus, Aquila chrysaetos, Circus aeruginosus*	11.2±13.7 (12.5 μm)	8.8–10.9 (9.9 μm)	not reported	Verma et al. (2017b), Juozaitytė-Ngugu et al. (2025)
*Sarcocystis lutrae*	*Lutra lutra, Vulpes lagopus, Vulpes vulpes, Nyctereutes procyonoides, Procyon lotor, Neovison vison, Martes foina, Meles meles, Mustela putorius*	*Haliaeetus albicilla*	11,3±12,3 μm	8,3±9,3 μm	not reported	Gjerde and Josefsen, (2015), Kirillova et al. (2018), Prakas et al. (2018a), Máca (2020), Máca and González-Solís (2022a)
*Sarcocystis arctica*	*V. lagopus, V. vulpes, Canis familiaris, Canis lupus*	*H. albicilla, B. buteo, M. milvus, Corvus corax, Corvus cornix*	10,6±12,7 μm	8,7±10,6 μm	not reported	Gjerde and Schulze (2014), Kirillova et al. (2018), Juozaitytė-Ngugu et al. (2021), Máca and González-Solís (2022b), Juozaitytė-Ngugu et al. (2025)
*Sarcocystis cheeli* (syn. *Isospora cheeli*)	not reported	*M. migrans*	8.33-10.47 µm (9.54 ± 0.387 µm)	5.89-7.32 µm (6.44 ± 0.3 µm)	not reported	Sharma and Shah (1990), Odening (1998)
*Sarcocystis calchasi*	*Columba livia, Zenaida asiatica, Streptopelia decaocto, Acryllium vulturinum, Dendrocopos major, Picus viridis, Nymphicus hollandicus, Polytelis alexandrae, Cacatua tenuirostris, Phalacrocorax penicillatus*	*A. gentilis, A. nisus, A. cooperii, B. jamaicensis*	11.9 μm	7.9 μm	not reported	Olias et al. (2010a), Rimoldi et al. (2013), Olias et al. (2014), Ziegler et al. (2018), Prakas et al. (2020a), Rogers et al. (2022), Šukytė et al. (2023)
*Sarcocystis columbae*	*Columba palumbus, Larus argentatus, Larus canus, Larus ridibundus*	*A. gentilis*, *A. nisus, A. cooperii, M. migrans, M. milvus, B. buteo, B. jamaicensis, F. tinnunculus, C. corax, C. cornix, B. bubo, G. fulvus, C. aeruginosus*	not reported	not reported	not reported	Olias et al. (2010a), Prakas et al. (2020a), Máca and González-Solís (2022b), Juozaitytė-Ngugu et al. (2025)
*Sarcocystis alectoributeonis*	*Alectoris chucar*	*B. buteo*	8,4±10,5 μm	7,0±8,4 μm	not reported	Pak et al. (1989b), Odening (1998)
*Sarcocystis halieti*	*Phalacrocorax carbo*, *Nannopterum brasilianum*, *L. argentatus, Larus dominicanus, L. canus,Chroicocephalus ridibundus,Puffinus puffinus,Accipiter striatus*, *A. cooperii*, *A. nisus, A. chrysaetos, Sturnus vulgaris*, *Athene noctua*, *C. aeruginosus*, *M. migrans, C. corax, C. cornix*, *Gypaetus barbatus,Stercorarius chilensis,* the great cormorant and possible other birds	*H. albicilla*, *A. nisus*, *A. gentilis*, *A. cooperi, G. barbatus*, *M. milvus, M. migrans, B. lineatus, B. jamaicensis, B. buteo, A. cooperii, C. aeruginosus, F. tinnunculus, G. fulvus, Pica pica, C. corax, C. cornix, Coloeus monedula*	16.0±17.0 μm (from *H. albicilla*) 12,8±15,8 μm (from *M. milvus*)	10.5±11.2 μm (from *H. albicilla*) 8,6±10,9 μm (from *M. milvus*)	not reported	Gjerde et al. (2018), Llano et al. (2022), Prakas et al. (2020a), Prakas et al. (2021), Juozaitytė-Ngugu et al. (2022), Máca and González-Solís (2022a, b), Rogers et al. (2022), Sato et al. (2022), Prakas et al. (2024a), Baker et al. (2025), Sazmand et al. (2025), Juozaitytė-Ngugu et al. (2025)
*Sarcocystis lari*	*Larus marinus, L. argentatus*	*H. albicilla, A. gentilis*	16.0±17.0 μm	10.5±11.2 μm	not reported	Prakas et al. (2014); Gjerde et al. (2018), Prakas et al. (2020b), Llano et al. (2022), Máca and González-Solís (2022a), Šukytė et al. (2023)
*Sarcocystis fulicae*	*Fulia atra*	not reported	not reported	not reported	not reported	Prakas et al. (2018b)
*Sarcocystis accipitris*	*Serinus canaria*	*A. gentilis*	15±17 μm	13±15 μm	not reported	Černá and Kvašňovská (1986), Odening (1998)
*Sarcocystis turdusi*	*Turdus merula, Turdus pilaris, Turdus philomelos, Erithacus rubecula*	*A. gentilis*, *A. nisus, A. striatus, B. buteo, B. jamaicensis, A. cooperii, P. pica, C. corax, C. cornix, C. monedula*	not reported	not reported	not reported	Kutkienė et al. (2012), Prakas et al. (2020a), Juozaitytė-Ngugu et al. (2025)
*Sarcocystis cooperii*	*Pitangus sulphuratus*	*A. cooperii*	not reported	not reported	not reported	Llano et al. (2025)
*Sarcocystis falcatula* (syn. *Isospora boughtoni*, *Sarcocystis corderi, Sarcocystis debonei*, *Sarcocystis oliverioi*, *Sarcocystis jacarinae*, *Sarcocystis setophagae, Balbiania falcatula*)	Species of Passeriformes; Cuculiformes; Coraciiformes, Suliformes, Charadriiformes, Pelecaniformes, Columbiformes; Sphenisciformes, Anseriformes; Accipitriformes; Strigiformes, Falconiformes; Psittaciformes and Piciformes birds [*Molothrus ater*, *Molothrus bonariensis*, *Pheucticus ludovicianus*, *Quiscalus mexicanus*, *Quiscalus quiscula*, *Passer domesticus*, *Poephila guttata*, *S. canarius*, *Merops nubicus*, *Cyclarhis gujanensis*, *Tachyphonus coronatus*, *Ramphocelus bresilius*, *Cacicus haemorrhous*, *Guira guira*, *Sula leucogaster, L. dominicanus, N. brasilianum, Stercorarius skua, Phimosus infuscatus, C. livia*, *Cyanoliseus patagonus*, *Goura victoria*, *Gallicolumba luzonica*, *Patagioenas picazuro*, *Leptotila rufaxilla*, *Zenaida auriculata*, *Zenaida macroura*, *Eudyptes chrysocome, Spheniscus demersus, Anas* sp., *A. chrysaetos*, *Haliaeetus leucocephalus*, *Parabuteo unicinctus*, *Rupornis magnirostris*, *B. jamaicensis*, *Bubo virginianus, Megascops choliba, Megascops asio S. varia, Melopsittacus undulatus*, *Cacatua alba*, *Psittacula krameri*, *Psittacus erithacus*, *Trichoglossus moluccanus*, *Brotogeris tirica*, *Pionus maximiliani*, *Amazona aestiva*, *Celeus flavescens*, *Dryocopus lineatus*, *Ramphastos dicolorus*, *Pteroglossus bailloni*, (some of them experimentally)].	*Didelphis virginiana, Didelphis aurita, Didelphis albiventris, Didelphis marsupialis*	9,6±12,0 (11,2 μm)	6,0±8,4 (7,4 μm)	1,5	Levine and Tadros (1980), Box et al. (1984), Odening (1998). Wünschmann et al. (2010); Llano et al. (2022), Sato et al. (2022), Šukytė et al. (2023), Baker et al. (2025)
*Sarcocystis wobeseri*	*Anas platyrhynchos, Branta leucopsis, L. argentatus, H. albicilla*	*A. gentilis, A. nisus, B. buteo*	not reported	not reported	not reported	Kutkienė et al. (2010), Prakas et al. (2011), Verma et al. (2017a), Shadbolt et al. (2021), Máca and González-Solís (2022b), Šukytė et al. (2023), Šukytė et al. (2024)
*Sarcocystis nontenella*	*B. buteo*	not reported	not reported	not reported	not reported	Levine and Tadros (1980), Odening (1998)
*Sarcocystis cornixi*	*C. cornix, C. monedula*	*A. gentilis*, *A. nisus, M. migrans, M. milvus, B. buteo, F. tinnunculus, P. pica, C. corax, C. cornix, Corvus frugilegus, B. bubo, Garrulus glandarius, C. aeruginosus*	not reported	not reported	not reported	Kutkienė et al. (2009), Mayr et al. (2016), Prakas et al. (2020a), Juozaitytė-Ngugu et al. (2022), Juozaitytė-Ngugu et al. (2025)
*Sarcocystis corvusi*	*C. monedula*	not reported	not reported	not reported	not reported	Prakas et al. (2013)
*Sarcocystis kutkienae*	*C. corax, C. cornix, P. pica*	*A. gentilis, M. migrans, B. buteo, P. pica, C. corax, C. cornix, C. frugilegus, G. glandarius, C. monedula*	not reported	not reported	not reported	Prakas et al. (2020a), Juozaitytė-Ngugu et al. (2022), Šukytė et al. (2023), Juozaitytė-Ngugu et al. (2025)

### Brief taxonomic review

Based on the findings presented herein, we conducted a comprehensive survey of *Sarcocystis* species reported to involve Accipitriformes in their life cycles to date (Table 1). In this survey, the genus *Frenkelia* was treated as a junior synonym of *Sarcocystis*, following the taxonomic interpretations proposed by Odening (1998) and Modrý et al. (2004). An important nomenclatural issue arises from the synonymization of *F. microti* Biocca, 1965, the species upon which the genus *Frenkelia* was originally erected. Under standard taxonomic practice, synonymization would naturally result in the combination *Sarcocystis microti*, retaining the original specific name. However, as highlighted by Modrý et al. (2004), this binomen had already been assigned to *S. microti*, a distinct species parasitizing cricetid rodents, thereby creating a case of homonymy. To avoid this issue, Odening (1998) synonymized *F. microti* with *Isospora buteonis*. Nevertheless, the original description of *I. buteonis* was based exclusively on oocysts shed by raptors belonging to four species from two different orders (Accipitriformes and Strigiformes), without adequate host specificity. Given that exogenous stages (oocysts and sporocysts) of Sarcocystidae exhibit substantial morphological uniformity and do not allow reliable species identification, and considering the absence of biological or experimental confirmation, Modrý et al. (2004) argued that this synonymization was also inappropriate. Consequently, *Sarcocystis buteonis* should be regarded as a *species inquirenda*, potentially representing a composite description based on more than one species. To resolve this nomenclatural impasse, Modrý et al. (2004) proposed the replacement name *Sarcocystis jaypeedubeyi* for *F. microti*. Additional synonyms attributed to this species include *Toxoplasma microti* and *Toxoplasma glareolus* (Odening, 1998).

Following the synonymization of *Frenkelia*, the species *F. glareoli* was transferred without major complications to *Sarcocystis glareoli* (Odening, 1998; Mugridge et al., 1999). Both *S. jaypeedubeyi* and *S. glareoli* utilize rodents as intermediate hosts and hawks as definitive hosts, and both species are extensively characterized in the literature with respect to host range, life cycle, ecological traits, and the morphology and morphometry of cysts and oocysts/sporocysts. Molecular and phylogenetic analyses further support the conclusion that *Frenkelia* does not warrant recognition as a separate genus. Other synonyms associated with these taxa include *Toxoplasma glareoli* and *F. clethrionomyobuteonis* (Odening, 1998). It should be noted that some authors continue to treat *Frenkelia* as a subgenus of *Sarcocystis*, as exemplified by Verma et al. (2017a), who referred to these taxa as *Sarcocystis (Frenkelia) glareoli* and *Sarcocystis (Frenkelia) microti*.

In addition to *S. jaypeedubeyi* and *S. glareoli*, other species completing life cycles involving rodents as intermediate hosts and hawks as definitive hosts include *Sarcocystis citellibuteonis*, *Sarcocystis jamaicensis*, and *Sarcocystis strixi*. For *S. citellibuteonis*, Pak et al. (1989a) provided detailed morphometric data, reporting sporocyst dimensions averaging 10 × 12.5 μm. In the case of *S. jamaicensis*, the intermediate host was identified through experimental infection of IFN-γ knockout mice, whereas the definitive host was naturally infected; the description of sporocysts is clear and well documented (Odening, 1998; Verma et al., 2017a). *Sarcocystis strixi* was initially described by Verma et al. (2017b) from the owl *Strix varia* as its natural definitive host, using IFN-γ knockout mice as experimental intermediate hosts. Subsequently, Juozaitytė-Ngugu et al. (2025) expanded the host spectrum of *S. strixi* to include Accipitriformes as definitive hosts. Although other *Sarcocystis* species described from owls may also involve Accipitriformes, only *S. strixi* was included here, as it is the sole species listed in the most recent comprehensive survey by Juozaitytė-Ngugu et al. (2025). Notably, the morphometric descriptions of *S. jamaicensis* and *S. strixi* are identical (Table 1), reinforcing the notion that oocyst and sporocyst morphometry alone is insufficient for reliable species discrimination.

Regarding predator–prey cycles involving mammals and raptors, *Sarcocystis arctica* and *Sarcocystis lutrae* merit particular attention. *Sarcocystis lutrae* was initially described from *Lutra lutra*, with its definitive host remaining unknown (Gjerde & Josefsen, 2015). Phylogenetic analyses later indicated that the Arctic fox *Vulpes lagopus* could also serve as an intermediate host, reflecting its close relationship with *S. arctica* (Gjerde & Schulze, 2014). Based on phylogenetic placement and the predatory ecology of potential hosts, Gjerde and Josefsen (2015) hypothesized the white-tailed eagle *Haliaeetus albicilla* as the definitive host of *S. lutrae*. This hypothesis was subsequently supported by Kirillova et al. (2018), who identified both *S. lutrae* and *S. arctica* in *Vulpes vulpes*. Further evidence was provided by Máca (2020), who detected sarcocysts of *S. lutrae* in *Nyctereutes procyonoides* and *Procyon lotor*, and by Prakas et al. (2018a), who reported *S. lutrae* in several mustelid species. Definitive confirmation of *H. albicilla* as the definitive host of *S. lutrae* was provided by Máca and González-Solís (2022a), who also supplied the first detailed morphometric descriptions of the species. In a subsequent study, Máca and González-Solís (2022b) elucidated the life cycle of *S. arctica*, likewise identifying *H. albicilla* as its definitive host, with further definitive hosts added by Juozaitytė-Ngugu et al. (2025).

Another taxon subject to reclassification is *Sarcocystis cheeli*, originally described as *Isospora cheeli*. Sharma and Shah (1990) later recovered oocysts from *Milvus migrans* that matched the original description and illustrations of *I. cheeli*. Based on the absence of a Stieda body in the sporocysts, these authors reassigned the species to *Sarcocystis*. Experimental infections of rodents were unsuccessful, leaving the intermediate host unknown.

As discussed previously, two species complete their life cycles between Columbiformes and Accipitriformes: *S. calchasi* and *S. columbae*. Their host associations, molecular characteristics, and morphometric data are well documented, except for *S. columbae*, for which oocyst/sporocyst measurements remain unknown due to identifications being based exclusively on molecular data (Olias et al., 2010a,b). Olias et al. (2011) recovered sporocysts from *Accipiter* hawks suspected of co-infection with *S. calchasi*, *S. columbae*, and *Sarcocystis* sp. ex *A. nisus*, reporting average dimensions of approximately 8.0 × 13 μm, but were unable to assign these measurements to a specific species without molecular confirmation. Subsequent studies demonstrated that *S. columbae* also utilizes Charadriiformes as intermediate hosts (Prakas et al., 2020b; Juozaitytė-Ngugu & Prakas, 2023). Rogers et al. (2022) further expanded the host range of *S. calchasi* by identifying *Accipiter cooperii* and *B. jamaicensis* as definitive hosts, supporting the notion that a single *Sarcocystis* species may occupy extensive geographic ranges and exploit multiple definitive hosts. Additional columbid intermediate hosts for *S. calchasi* were reported by Šukytė et al. (2023).

Another well-defined cycle involves *Sarcocystis alectoributeonis*, which uses *Alectoris chukar* as an intermediate host and *Buteo buteo* as a definitive host; its morphology and measurements are well established (Pak et al., 1989b).

*Sarcocystis lari* and *S. halieti* are species that use aquatic birds as intermediate hosts and raptors as definitive hosts. *Sarcocystis lari* was originally described by Prakas et al. (2014) without identification of its definitive host. Subsequently, Gjerde et al. (2018) detected oocysts of this species in the white-tailed sea eagle *H. albicilla*. In the same study, the authors described *S. halieti* as a new species and also detected *Sarcocystis truncata* in fecal samples. Molecular characterization was performed from mucosal scrapings containing oocysts, sequencing four DNA regions (18S rRNA, 28S rRNA, ITS1, and *cox1*). Despite the detection of three species, only *S. lari* and *S. halieti* were considered to complete a natural life cycle between aquatic birds and sea eagles. *Sarcocystis truncata*, whose intermediate host is the red deer *Cervus elaphus* and whose definitive hosts are likely felids based on phylogenetic placement and prevalence, was interpreted as a case of pseudoparasitism, probably resulting from ingestion of infected prey. For this reason, *S. truncata* was not included in our table. Although oocysts and sporocysts were observed, their morphometric characterization was ambiguous. Most oocysts were thin-walled and sporulated, measuring on average approximately 22 × 16 µm, whereas sporocysts (free or within oocysts) measured approximately 16.5 × 11 µm. A small number of markedly smaller forms were also detected, with one oocyst measuring approximately 14.5 × 11.5 µm and sporocysts averaging approximately 11.5 × 8 µm; these were attributed to *S. truncata*. Because the authors did not clearly assign morphometric data to each species, *S. lari* and *S. halieti* are considered morphometrically indistinguishable. Indeed, these two species can only be reliably differentiated using ITS1 sequences (Gjerde et al., 2018).

Subsequent studies expanded the known host range of *S. halieti*. Juozaitytė-Ngugu et al. (2022) demonstrated that corvids can act as intermediate hosts, and Máca and González-Solís 2022a, 2022b) reported *Milvus milvus* as a definitive host and *Sturnus vulgaris* as an intermediate host. Sporocysts recovered from *M. milvus* were on average slightly smaller than those reported from *H. albicilla*, reinforcing the limited reliability of morphometry alone for species discrimination. Muscle cysts attributed to *S. halieti* were also identified in *M. migrans* and *Circus aeruginosus* (Prakas et al., 2021), demonstrating that raptors may function not only as definitive but also as intermediate hosts. Additional intermediate hosts of *S. halieti* include *Gypaetus barbatus* and, based on molecular similarity, the scavenging vultures *Coragyps atratus* and *Vultur gryphus*, whose sarcocysts clustered with *S. lari* and *Sarcocystis* sp. ex *Larus dominicanus* (Prakas et al., 2024a; Llano et al., 2025). These findings suggest that necrophagous Cathartiformes may participate as intermediate hosts in the life cycle of this lineage.

*Sarcocystis fulicae*, described from *Fulica atra* (Gruiformes), lacks a confirmed definitive host; however, its phylogenetic position among species cycling through Accipitriformes strongly suggests raptors such as *C. aeruginosus* or *H. albicilla* as likely definitive hosts (Prakas et al., 2018b). Accordingly, this species was included in our survey.

Several *Sarcocystis* species circulate between Passeriformes and Accipitriformes, including *Sarcocystis accipitris*, *S. turdusi*, and *Sarcocystis cooperii*. *Sarcocystis accipitris* is well characterized, with *Serinus canaria* as an experimentally confirmed intermediate host and *A. gentilis* as a natural definitive host; sporocysts were described by Černá and Kvašňovská (1986). *Sarcocystis turdusi*, initially described only from muscle cysts in *Turdus merula*, was later shown by phylogenetic inference and molecular detection to use *A. gentilis* and *A. nisus* as definitive hosts (Mayr et al., 2016), although oocyst and sporocyst morphometry remains unknown. Additional intermediate and definitive hosts have since been reported (Juozaitytė-Ngugu et al., 2025). The possibility that *S. turdusi* is synonymous with *Sarcocystis turdi* remains unresolved. *Sarcocystis cooperii* was described based on molecular data from muscle cysts in *Pitangus sulphuratus*, showing 99.9% similarity with *Sarcocystis* sp. ex *A. cooperii*, thereby identifying *A. cooperii* as the definitive host despite the absence of direct sampling. Consequently, morphometric data for exogenous stages are unavailable.

For several decades, Accipitriformes have been proposed as intermediate hosts of *Sarcocystis* spp. (Krone et al., 2000; Olson et al., 2007). Among these species, *S. falcatula* stands out for exhibiting the widest diversity of avian intermediate hosts, encompassing species from Accipitriformes, Columbiformes, Piciformes, Psittaciformes, Passeriformes, among others (Table 1). According to the literature, opossums of the genus *Didelphis* are the only confirmed definitive hosts of this species. Given the remarkably broad range of avian intermediate hosts, some authors have suggested that *S. falcatula* may incorporate paratenic hosts into its life cycle Llano et al. (2022).

In the latter study, parasitism by *Sarcocystis* sp. was associated with meningoencephalitis in a bald eagle *Haliaeetus leucocephalus*. Immunohistochemical analyses showed a positive reaction for *S. neurona*, whereas reactions for *T. gondii* and *Neospora caninum* were negative. Nevertheless, the inclusion of Accipitriformes as intermediate hosts of *S. neurona* cannot be confirmed. As emphasized by the authors, *S. neurona* is phylogenetically related to *S. falcatula,* both sharing opossums as definitive hosts, but differs in host range: *S. neurona* infects mammals but not birds, whereas *S. falcatula* infects birds but not mammals. Thus, although *S. neurona* causes neurological disease in several mammalian species, there are no confirmed avian infections. Antigenic cross-reactivity between *S. neurona*, *S. falcatula*, and possibly other *Sarcocystis* species cannot be excluded. Moreover, as molecular methods were not employed and the specific parasite could not be determined, *S. neurona* is not considered involved in parasitism of Accipitriformes.

Another species involving Accipitriformes as intermediate hosts is *Sarcocystis wobeseri*. This species was originally described with Anseriformes as intermediate hosts and an unknown definitive host (Kutkienė et al., 2010). Later, *Larus argentatus* was identified as an additional intermediate host (Prakas et al., 2011), demonstrating low host specificity. Subsequent studies confirmed *L. argentatus* as an intermediate host of *S. wobeseri*, *S. columbae*, *S. halieti*, and *S. lari* (Prakas et al., 2020b). Phylogenetic analyses grouped *S. wobeseri* with species that use raptors as definitive hosts (e.g., *S. calchasi*, *S. columbae*, *S. cornixi*), suggesting a similar life cycle. However, Shadbolt et al. (2021) molecularly identified *S. wobeseri* in skeletal and cardiac muscle of *H. albicilla*, characterizing this raptor as a new intermediate host. The definitive host of *S. wobeseri* remains unknown, which is noteworthy given that *H. albicilla* is a confirmed definitive host of other *Sarcocystis* species.

Several studies have also demonstrated that *S. halieti* uses Accipitriformes as intermediate hosts (Prakas et al., 2021). Supporting this, *S. halieti* was identified in *Accipiter striatus*, suggesting that hawks can act as both intermediate and definitive hosts (Llano et al., 2022). This dual role was further corroborated by Sazmand et al. (2025), who genetically confirmed *S. halieti* in skeletal muscle of *A. nisus* using four nuclear and mitochondrial markers (18S rRNA, 28S rRNA, ITS1, and *cox1*), demonstrating that this species can function as both definitive and intermediate host.

*Sarcocystis nontenella*Levine and Tadros (1980) was described using *B. buteo* as an intermediate host. Levine and Tadros (1980) corrected the original name *Sarcocystis tenella* due to homonymy with *S. tenella* infecting sheep. Information on this species remains scarce; its definitive host and morphometric data of oocysts/sporocysts are still unknown.

Among corvid-associated species, *S. cornixi*, *Sarcocystis corvusi*, and *Sarcocystis kutkienae* have been described. Only *S. cornixi* has Accipitriformes confirmed as definitive hosts, but phylogenetic evidence suggests raptor involvement for the remaining species as well. *Sarcocystis cornixi* was first described in *Corvus cornix* (Kutkienė et al., 2009), and later *Accipiter* spp. were identified as definitive hosts based on molecular characterization of exogenous stages, leaving oocyst/sporocyst measurements unknown (Mayr et al., 2016; Prakas et al., 2020a). *Coloeus monedula* was later identified as an additional intermediate host (Juozaitytė-Ngugu et al., 2022).

*Sarcocystis corvusi*, described from *C. monedula*, remains known only from its intermediate host, with no data on exogenous stages (Prakas et al., 2013). Its close phylogenetic relationship with *S. columbae*, *S. calchasi* and *S. wobeseri* suggests a life cycle involving raptors. Similarly, *S. kutkienae* clusters phylogenetically with several raptor-associated *Sarcocystis* spp., and although its definitive host remains unknown, birds of prey are presumed to fulfill this role (Prakas et al., 2020a).

Llano et al. (2025) highlighted that Juozaitytė-Ngugu et al. (2025) confirmed *B. buteo*, *A. gentilis* and *A. nisus* as definitive hosts of *Sarcocystis* sp. ex *Corvus corax*, reinforcing the view that *Sarcocystis* spp. infecting corvids commonly use Accipitriformes as definitive hosts. Additionally, *C. cornix* and *Pica pica* were identified as intermediate hosts of *S. kutkienae* (Juozaitytė-Ngugu et al., 2022).

Similar to birds of prey, corvids (omnivorous birds) have been shown to act as both intermediate and definitive hosts of *Sarcocystis* spp. (Juozaitytė-Ngugu et al., 2021; Sazmand et al., 2025). Juozaitytė-Ngugu et al. (2021) examined intestinal scrapings from several corvid species and molecularly identified eleven *Sarcocystis* spp.: *S. columbae*, *S. cornixi*, *S. halieti*, *S. kutkienae*, *S. lari*, *S. turdusi*, *S. wobeseri*, *S. arctica*, *S. lutrae*, *Sarcocystis ovalis* and *Sarcocystis oviformis*. Of these, only the first nine are included in our table due to their confirmed association with raptors. The authors emphasized that three of these species use corvids as intermediate hosts, indicating that, as observed in Accipitriformes, corvids may function as both intermediate and definitive hosts for the same *Sarcocystis* species. Oocysts observed in the intestinal mucosa measured approximately 20.5 × 19.5 μm, although free sporocysts were not detected, and these measurements were not assigned to specific species. The examined corvids included *C. cornix*, *C. corax*, *C. monedula*, *Corvus frugilegus*, *P. pica* and *Garrulus glandarius*. All eleven *Sarcocystis* spp. were detected in *C. cornix*, and *S. halieti* was the most frequently recorded species, occurring in *C. cornix*, *C. corax*, *C. monedula*, and *P. pica*. *C. cornix* and *C. corax* showed significantly higher infection frequencies, consistent with their scavenging behavior. Nevertheless, the authors caution that the detection of *Sarcocystis* DNA in intestinal or fecal samples does not conclusively demonstrate definitive host status, as pseudoparasitism may occur. Oocysts were observed microscopically in approximately 47% of samples, whereas molecular detection reached about 85%, suggesting that some DNA may originate from ingested tissues containing sarcocysts or from environmental contamination. Consequently, experimental life-cycle studies are considered necessary. Despite these limitations, this information was included in our table in accordance with recent syntheses (e.g., Juozaitytė-Ngugu et al., 2025), but should be interpreted with caution.

Although *Sarcocystis* is typically heteroxenous, some species using rodents and reptiles as intermediate hosts (*Sarcocystis cymruensis*, *Sarcocystis dugesii*, *Sarcocystis galotiae*, *Sarcocystis muris*, *Sarcocystis simonyi* and *Sarcocystis stehlinii*) exhibit both diheteroxenous and dihomoxenous life cycles, enabling transmission via cannibalism. This biological flexibility may explain why corvids and Accipitriformes can serve as both intermediate and definitive hosts for certain *Sarcocystis* spp.

Finally, Šukytė et al. (2023) investigated intestinal scrapings of *A. gentilis* and *A. nisus*, finding high prevalences of oocysts/sporocysts. Molecular analyses identified *S. columbae*, *S. halieti*, *S. turdusi*, *S. wobeseri*, *S. calchasi*, *S. cornixi*, *S. kutkienae* and *S. lari* in *A. gentilis*, whereas only *S. columbae*, *S. halieti*, *S. turdusi* and *S. wobeseri* were detected in *A. nisus*. A genetically distinct lineage (*Sarcocystis* sp. 23LTAcc), closely related to *S. calchasi*, was also identified in *A. gentilis*. Sporocysts measured approximately 13 × 9 μm in *A. gentilis* and approximately 12 × 8 μm in *A. nisus*, although these measurements were not linked to specific species. Similarly, Prakas et al. (2024c) reported sporocysts measuring approximately 12.5 × 8.5 μm in *Buteo lagopus* and approximately 13 × 9 μm in *B. buteo*, but without species attribution. Šukytė et al. (2024) found sporocysts in *B. buteo* averaging approximately 14.5 × 11 μm, while molecular analyses identified *S. glareoli*, *S. cornixi*, *S. halieti*, *S. kutkienae*, *S. turdusi* and *S. wobeseri*, as well as three potentially new species (Sarcocystis sp. Rod3–Rod5). Juozaitytė-Ngugu et al. (2025) conducted a comprehensive molecular survey of raptors from Accipitridae, Falconidae, and Strigidae, analyzing 40 individuals and confirming twelve known *Sarcocystis* species and three genetically novel taxa in their intestines. These findings substantially expand knowledge of definitive hosts among Accipitriformes and have been incorporated into our table.

## Conclusions

In conclusion, the findings of the present study contribute to a broader understanding of *Sarcocystis* spp. in natural environments, highlighting the need for further investigations to clarify the role of Neotropical birds as both intermediate and definitive hosts, particularly in Southeastern Brazil. We further emphasize that an integrative taxonomic approach is essential for the accurate identification of coccidia for which clear morphological taxonomic characters are lacking. Accordingly, the combined application of molecular analyses, comparative morphology and morphometry, host identification, infection site assessment, and histopathological examination is crucial for the reliable delineation and identification of Sarcocystidae species.
